# Effectiveness of micronized purified flavonoid fraction in early stage chronic venous disease—Clinical, Etiological, Anatomical, and Pathophysiological C0s-C1: A systematic review and meta-analysis

**DOI:** 10.1016/j.jvsv.2026.102558

**Published:** 2026-06-22

**Authors:** Bouskela Eliete, Zolotukhin Igor, Amore Miguel, Ministro Augusto, Blanc-Guillemaud Vanessa, Onselaer Marie-Blanche, Serifou Soumeya, Yaltirik Hurrem Pelin, Sirlak Mustafa, Mansilha Armando

**Affiliations:** a Department of Physiological Sciences, State University of Rio de Janeiro, Rio de Janeiro, Brazil; b Department of Fundamental and Applied Research in Cardiovascular Surgery, Pirogov Russian National Research Medical University, Moscow, Russia; c Phlebology and Lymphology Unit, Cardiovascular Surgery Division, Central Military Hospital, Buenos Aires, Argentina; d Centro Cardiovascular da Universidade de Lisboa, Faculdade de Medicina da Universidade de Lisboa, Lisboa, Portugal; e Unidade Local de Saúde de Santa Maria, Lisboa, Portugal; f Servier Affaires Médicales, Suresnes, France; g Soladis Clinical Studies, Roubaix, France; h Soladis, Lyon, France; i Department of Cardiovascular Surgery, Ankara University, Faculty of Medicine, Ankara, Turkey; j Faculty of Medicine, University of Porto, Porto, Portugal

**Keywords:** Chronic venous disease, C0s-C1, Meta-analysis, Micronized purified flavonoid fraction, Venous symptoms

## Abstract

**Objective:**

Chronic venous disease is a progressive disease that often begins with venous symptoms even without visible signs. This systematic review and meta-analysis aimed to evaluate the effectiveness of micronized purified flavonoid fraction (MPFF) in patients with early stage chronic venous disease (Clinical, Etiological, Anatomical, and Pathophysiological C0s-C1).

**Methods:**

A systematic search was conducted following the Preferred Reporting Items for Systematic Reviews and Meta-Analysis guidelines. MEDLINE, Embase, and Cochrane databases were searched from inception until November 2023. Prospective studies (randomized controlled trials and comparative, single-arm, and observational studies) assessing MPFF (1000 mg daily orally) in C0s-C1 patients were included. Outcomes included leg venous symptoms (pain, heaviness, and cramps) and quality of life (QoL). The data were analyzed using a random-effects meta-analysis model.

**Results:**

Five studies involving 411 patients were analyzed. The patients’ mean age was 40.1 (8.0) years, and 96.6% of them were female. Patients were classified as Clinical, Etiological, Anatomical, and Pathophysiological C1 (80%) and C0s (20%). MPFF significantly reduced pain intensity (mean change [MC], -2.3 cm; 95% confidence interval [CI], -3.1 to -1.6), reduced heaviness (MC, -2.8 cm; 95% CI, -3.5 to -2.1), and improved QoL (MC, -16.0; 95% CI, -20.7 to -11.3). Two studies reported complete resolution of symptoms for pain (100.0%), heaviness (87.9%), and cramps (97.8%). Two studies had a high risk of bias, and the heterogeneity level was frequently high among studies.

**Conclusions:**

The present meta-analysis reinforces the evidence supporting MPFF effectiveness in alleviating venous leg symptoms and improving QoL in C0s-C1 patients and supports the use of MPFF treatment in early disease management.

**Article Highlights**

• **Type of Research:** Systematic review and meta-analysis
• **Key Findings:** Five studies were analyzed, including 411 patients with early stages of chronic venous disease (Clinical, Etiological, Anatomical, and Pathophysiological C0s-C1). Micronized purified flavonoid fraction significantly reduced pain intensity (mean change [MC], -2.3 cm; 95% confidence interval [CI], -3.1 to -1.6), heaviness (MC, -2.8 cm; 95% CI, -3.5 to -2.1), and improved quality of life (MC, -16.0 points; 95% CI, -20.7 to -11.3). Complete resolution of symptoms was reported for pain (100.0%), heaviness (87.9%), and cramps (97.8%).
• **Take Home Message:** This meta-analysis adds to the evidence supporting the effectiveness of micronized purified flavonoid fraction treatment in relieving venous leg symptoms and improving quality of life in patients with early stages of chronic venous disease.

Chronic venous disease (CVD) is defined as any morphological and functional abnormality of the venous system of long duration, manifested by symptoms or signs that indicate the need for investigation or care.^1^ CVD is extremely common, particularly in industrialized countries, and may progress in most cases.^2^, ^3^, ^4^ The disease is graded based on signs and symptoms according to the Clinical, Etiological, Anatomical, and Pathophysiological (CEAP) classification.^5^, ^6^, ^7^

In the earliest stages (CEAP C0s), patients have no visible clinical signs but report venous-related symptoms, such as pain or leg heaviness, which can be easily overlooked or attributed to normal fatigue or aging.^6^^,^^7^ Epidemiological studies estimate that early stage CVD (C0s-C1) affects 35.0% of the general population,^8^ and data from the Vein Consult Program indicated that 20.0% and 21.7% of patients with CVD screened by general practitioners were classified as C0s and C1, respectively.^9^

Although C0s-C1 patients are increasingly recognized, their clinical management remains inadequate.^10^^,^^11^ Many patients with venous symptoms do not seek medical attention, and even when they do, few are referred to specialists or receive appropriate treatment according to the international guidelines.^9^ This is reflected in the Vein Consult study, which showed that general practitioners consider only 25.3% of C0s and 74.5% of C1 patients as patients with CVD.^9^ Unfortunately, if left untreated, the early stages of CVD may progress to chronic venous insufficiency (CVI), thereby increasing the treatment burden and cost while reducing patients’ quality of life (QoL).^8^^,^^11^ In addition, CVD may be a manifestation of serious underlying vascular disease and is associated with an increased risk of cardiovascular disease and all-cause mortality.^12^^,^^13^

The 2022 European Society for Vascular Surgery clinical practice guidelines recommend considering venoactive drugs (VADs) for the reduction of venous symptoms in patients with symptomatic CVD from C0s, particularly in those who are not undergoing interventional treatment, are awaiting intervention, or have persistent symptoms and/or edema after intervention.^14^ In addition, Nicolaides et al^15^ reported that VADs may contribute to symptom improvement across all stages of CVD, including patients classified as CEAP C0s. Among VADs, micronized purified flavonoid fraction (MPFF), which contains 90.0% diosmin and 10.0% other active flavonoids (diosmetin, hesperidin, linarin, and isorhoifolin, expressed as hesperidin), has demonstrated both symptom relief and improvement in QoL.^14^^,^^15^ While most studies have evaluated the effects of MPFF across CEAP classes, as reflected in recent meta-analyses demonstrating reductions in key CVD symptoms (including leg pain, heaviness, swelling, and the sensation of edema) as well as objective signs such as lower-limb edema,^16^, ^17^, ^18^, ^19^ evidence addressing its role in patients at an early stage of the disease is limited. These studies were summarized in a recent review by Mansilha et al,^11^ which highlighted the rationale for early MPFF treatment through its beneficial effects on the underlying causes of the disease.

Given the limited data available on the management of patients with early stages of CVD, there is a need to evaluate interventions such as MPFF in these specific patients. This will strengthen the evidence base and guide clinical practice to improve patient outcomes in this population. Therefore, the aim of this work is to synthesize the current evidence on the effectiveness of MPFF in alleviating venous symptoms and improving health-related QoL in adults with C0s-C1 CVD by conducting a single-group meta-analysis that combines studies with different designs.

## Methods

### Protocol and registration

This systematic review and meta-analysis followed the 2020 Preferred Reporting Items for Systematic Reviews and Meta-Analysis statements.^20^ The review protocol was registered in PROSPERO (CRD42024495344).

### Search strategy

The following databases were searched from inception to November 2023 for relevant studies: MEDLINE (through PubMed), Embase (through the Cochrane Central Register of Controlled Trials interface), and Cochrane databases.

The searches were based on the Patient, Intervention, Comparison, and Outcome search strategy. The population included patients (>18 years of age) suffering from any type of CVD, regardless of the diagnosis method, who were at an early stage of the disease defined according to the CEAP clinical classification as patients presenting with C0s to C1 disease (ie, symptomatic patients without visible signs or with telangiectasias/reticular veins, without varicose veins, edema, skin changes, or ulceration). The intervention studied was oral MPFF (1000 mg daily, either 500 mg twice daily or 1000 mg in one single intake) with at least 1 month of treatment. No comparison was required. The main outcomes were leg venous symptoms, including pain, heaviness, and cramps, as well as QoL.

The search strategy performed by a librarian combined Medical Subject Headings terms and free-text keywords using Boolean operators (Supplementary Table I, online only). In addition, a manual search was performed to identify potential articles missing from the initial electronic searches in Servier databases as well as from the reference lists of recent available reviews.

### Study selection

The inclusion criteria were any prospective study (randomized controlled trials [RCTs] and non-RCTs: ie, comparative, single-arm, and observational studies). Patients who had undergone venous procedural interventions (eg, endovenous ablation, sclerotherapy, etc) were excluded. No restrictions on language or publication year were applied during the search or screening process. Articles were translated into English when required.

### Outcome measures

Included studies evaluated any of the following outcome measures: leg symptoms (ie, pain, heaviness, and cramps) using a 10-cm visual analog scale assessed as a continuous variable when the mean (± standard deviation [SD] or standard error of the mean) was provided and/or as dichotomous variables when frequency was reported for symptom resolution; and QoL questionnaires using the ChronIc Venous Insufficiency Questionnaires (CIVIQ-14 and CIVIQ-20), the Aberdeen Varicose Veins Questionnaire, or Veines-QOL as a continuous variable when the mean score (± SD or standard error of the mean) was reported. Regarding the CIVIQ score, the Global Index Score (GIS) was considered, ranging from 0 (best QoL) to 100 (worst QoL).

### Data collection

Two reviewers (M.B.O. and C.D.) independently assessed the eligibility of studies identified by the searches and extracted relevant data from the articles. Consensus on the inclusion or exclusion of reports was reached through discussion between the reviewers. A third reviewer (V.B.G.) validated the final list of selected articles and the extracted data.

The extracted data included study characteristics (such as first author, date of publication, and study design), sample size, participant information (age, sex, body mass index [BMI], CVD etiology, and concomitant treatments), and the outcome measurements. For eligible RCTs, intention-to-treat data were preferentially extracted when available, while per-protocol data were used only when the intention-to-treat analyses were not reported.

### Assessment of the risk of bias

The risk of bias (RoB) was assessed independently by two reviewers (M.B.O. and C.D.) using the Cochrane RoB tools, with the RoB 2 tool used to evaluate RCTs^21^ and the ROBINS-I tool used^22^ for non-RCTs.

### Statistical analysis

Continuous data were expressed as mean changes (MCs) from baseline to the last postbaseline values with 95% confidence interval (95% CI) when outcomes were measured using the same scale across studies. Alternatively, when the outcomes were measured by different scales across studies, the standardized mean changes^23^ were used with the method of Algina and Keselman to estimate the variance.^24^ In cases where multiple postintervention time points were available, the last postbaseline value from each study was retained. When the change in continuous variables at study end was not reported, it was estimated from the initial and final values, assuming a correlation of 0.75.^25^ Qualitative data were expressed as the proportion of patients with complete symptom resolution.

Given the size of the combined samples, the individual treatment effect estimates were assumed to be normally distributed. A random-effects meta-analysis model was employed, adopting the restricted maximum likelihood method to estimate the pooled effect size.

If more than one treatment arm in a study met the inclusion criteria, data from these arms were pooled together prior to analysis. Heterogeneity across studies was assessed using key metrics: Cochran’s *Q* test, I-squared (*I*^2^) statistic, and tau-squared (τ^2^).^26^^,^^27^ Missing data were not imputed. Missing SDs were either derived or imputed in accordance with the Cochrane Handbook.^28^ Subgroup analyses were performed according to study design (RCTs vs non-RCTs) and treatment duration (≤2 months, 3 to 5 months, and ≥6 months). Sensitivity analysis was conducted to assess the robustness of the results by excluding studies identified with a high RoB. Statistical analyses were performed using R statistical software (version 3.6) with the *metafor* package.^29^

## Results

### Study selection

**Fig 1:**
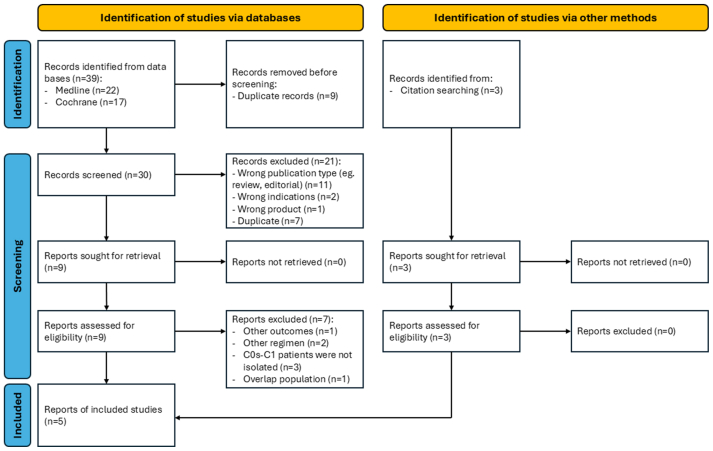
Preferred Reporting Items for Systematic Reviews and Meta-Analysis (*PRISMA*) flow diagram of study selection.

Forty-two publications were retrieved from databases and manual searches (Fig 1). Based on titles and abstracts, nine duplicates between databases and 21 other reports were excluded. Full-text analysis led to the exclusion of seven additional reports. Ultimately, five publications were included in the meta-analysis (Supplementary Table II, online only). One study was an RCT considered to have a low RoB^30^ (Supplementary Fig, *A*, online only). Four studies were non-RCTs,^31^, ^32^, ^33^, ^34^ two of which raised some concerns,^33^^,^^34^ and two studies were considered to have a high RoB^31^^,^^32^ (Supplementary Fig, *B*, online only). Among these, one study was reported as an abstract, with insufficient information available for evaluation.^31^ Another study presented confounding bias because of the absence of exclusion criteria or a washout period for VADs.^32^

### Study description

The studies included in the analysis enrolled a total of 717 participants, of whom 529 patients belonged to the C0s-C1 CEAP class. Of these, 411 were treated with MPFF 1000 mg daily (Supplementary Table II, online only).

With the exception of one international multicenter study,^30^ the remaining studies were monocentric and primarily conducted in Russia, with three studies published by the same first author. One publication was a post hoc analysis from a double-blinded RCT comparing two treatments with MPFF 1000 mg per day over 8 weeks (2 months).^30^ Three publications were open single-arm studies^31^, ^32^, ^33^ assessing the effectiveness of MPFF 1000 mg once daily or MPFF 500 mg twice daily after a treatment duration of 2 months,^32^ 3 months,^33^ or 6 months.^31^ One publication^34^ was an open-label study assessing treatment in different populations, including one arm comprising C0s-C1 patients with situational great saphenous vein (GSV) reflux treated with MPFF 1000 mg daily for 3 months (Supplementary Table II, online only).

### Baseline characteristics

Overall, the mean (SD) age of participants was 40.1 (8.0) years. The proportion of female participants was 96.6% (Supplementary Table III, online only), with three studies including only women.^32^, ^33^, ^34^ BMI was reported in two studies,^30^^,^^31^ totaling 286 participants with a mean BMI of 23.9 (3.1) kg/m^2^. In the RCT,^30^ approximately 65% of patients with C0s-C1 disease presented with reflux. All patients had transitory reflux in the studies by Tsukanov et al.^32^, ^33^, ^34^ In Lugli et al.^31^ reflux was observed at the microvalve level of visible superficial veins and saphenous tributaries down to the second generation.

According to the CEAP classification, the majority of the population was classified as C1 (80%), whereas the remaining 20% were classified as C0s. One study included only women classified as C0s,^32^ and one study included only women classified as C1.^33^ The etiology of CVD was reported as primary in all studies except one, in which the origin of the pathology was not identified^32^ (Supplementary Table II, online only). Concomitant treatments were reported exclusively in the RCT,^30^ which included hormonal therapy and angiotensin-converting enzyme inhibitors, while compression therapy was not used across studies.

### Venous leg symptoms

#### Pain

**Fig 2:**
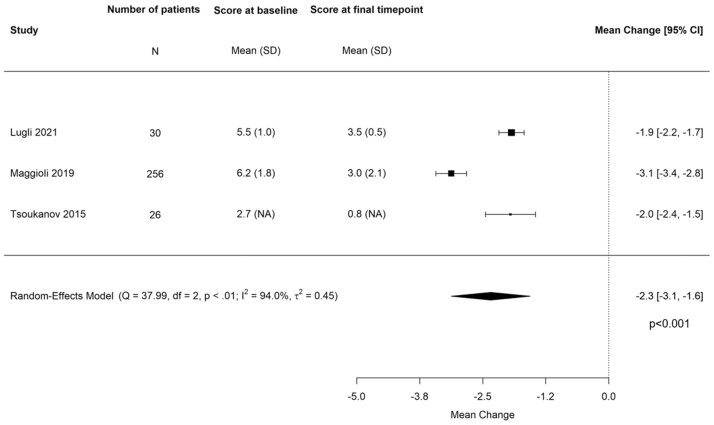
Leg pain symptom. Forest plot showing the pooled mean change (*MC*) of pain score intensity from baseline to the last postbaseline value. Error bars represent 95% confidence interval (*CI*). *df*, degrees of freedom; *I*^*2*^, *I*-squared; *N*, number; *NA*, not available; *Q*, Cochrane’s *Q* test statistics; *RE*, random effect; *SD*, standard deviation; *τ*^*2*^, tau-squared.

Pain score was reported in three studies (312 MPFF-treated participants).^30^, ^31^, ^32^ Pain intensity score from baseline to the last postbaseline value was significantly reduced, with a MC of −2.3 cm (95% CI, −3.1 to −1.6; *P* < .001). There was considerable heterogeneity across studies (*Q* = 37.99; *P* < .01; *I*^2^ = 94.0%; τ^2^ = 0.45) (Fig 2, Supplementary Tables IV and V, online only). Two studies were considered to have a high RoB,^31^^,^^32^ whereas only one study^30^ was considered to have a low RoB, precluding sensitivity analysis.

Complete resolution of pain symptoms was reported in two studies (29 participants).^33^^,^^34^ The pooled proportion of participants with complete resolution at the end of the studies was 100.0% (95% CI, 96.7-100.0%; *P* < .001), with no heterogeneity across studies (*Q* = 0.00; *P* = 1.00; *I*^2^ = 0.0%; τ^2^ = 0.00) (Supplementary Table VI, online only).

Subgroup analysis by study design including data from 256 MPFF-treated participants in a double-blind RCT^30^ showed a significant reduction in pain intensity score, with a MC of −3.1 cm (95% CI, −3.4 to −2.8; *P* < .001). In the non-RCT subgroup, data from two studies^31^^,^^32^ totaling 56 MPFF-treated participants showed a significant reduction, with a MC of −1.9 cm (95% CI, −2.2 to −1.7; *P* < .001), with no heterogeneity across studies (*Q* = 0.00; *P* = .94; *I*^2^ = 0.0%; τ^2^ = 0.00) (Supplementary Table IV, online only). A test for subgroup differences showed a significant result (*P* < .001).

Treatment duration subanalysis showed significant reductions in leg pain symptoms with MPFF treatment over different periods: 2 months, MC of −2.6 cm (95% CI, −3.7 to −1.4; *P* < .001); 3 months, MC of −1.3 cm (95% CI, −1.6 to −1.1; *P* < .001); and 6 months, MC of −1.9 cm (95% CI, −2.2 to −1.7; *P* < .001) (Supplementary Table IV, online only). No metaregression was implemented.

#### Leg heaviness

**Fig 3:**
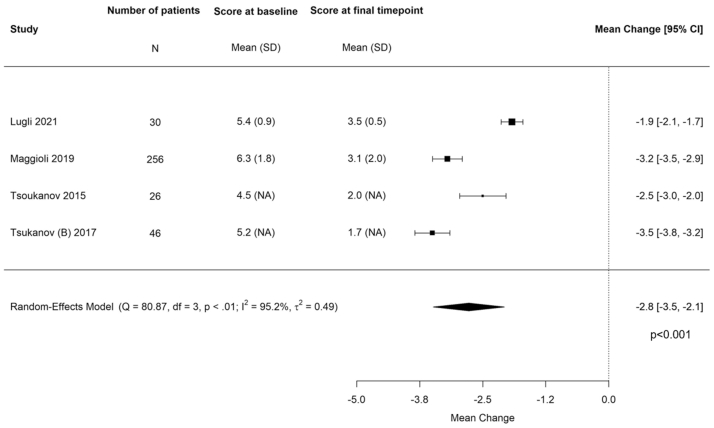
Leg heaviness symptom. Forest plot showing the pooled mean change (*MC*) of heaviness score intensity from baseline to the last postbaseline value. Error bars represent 95% confidence interval (CI). *df*, degrees of freedom; *I*^*2*^, *I*-squared; *N*, number; *NA*, not available; *Q*, Cochrane’s *Q* test statistics; *RE*, random effect; *SD*, standard deviation; *τ*^*2*^, tau-squared.

Leg heaviness scores were reported in four studies,^30^, ^31^, ^32^^,^^34^ totaling 358 MPFF-treated participants. A significant reduction was observed with a MC of −2.8 cm (95% CI, −3.5 to −2.1; *P* < .001). Considerable heterogeneity across studies was observed (*Q* = 80.87; *P* < .01; *I*^2^ = 95.2%; τ^2^ = 0.49). The sensitivity analysis did not change the results, with a MC of −3.3 cm (95% CI, −3.6 to −3.0; *P* < .001) (Fig 3, Supplementary Tables IV and VII, online only).

Complete resolution of heaviness symptoms was reported in two studies (99 participants).^33^^,^^34^ The pooled proportion of participants with complete resolution was 87.9% (95% CI, 80.8-93.6%; *P* < .001), with no heterogeneity across studies (*Q* = 0.13; *P* = .721; *I*^2^ = 0.0%; τ^2^ = 0.00) (Supplementary Table VIII, online only).

Subgroup analyses by study design showed significant reductions in both RCTs and non-RCTs, with a MC of −3.2 cm (95% CI, −3.5 to −2.9; *P* < .001) and −2.6 cm (95% CI, −3.6 to −1.7; *P* < .001), respectively. There was an important interstudy heterogeneity (*Q* = 58.23; *P* < .01; *I*^2^ = 95.7%; τ^2^ = 0.629) but no significant subgroup difference (*P* = .54) (Supplementary Table IV, online only).

Regarding treatment duration, data from two studies (282 MPFF-treated participants)^30^^,^^32^ showed a significant improvement in heaviness score after 2 months of treatment, with a MC of −2.9 cm (95% CI, −3.6 to −2.2; *P* < .001) and a substantial heterogeneity across studies (*Q* = 6.35; *P* = .01; *I*^2^ = 84.3%; τ^2^ = 0.21). Data from two studies (76 participants)^31^^,^^34^ showed a decrease in heaviness intensity of −2.2 cm (95% CI, −4.8 to −0.5) after 3 months of treatment, but this was not significant (*P* = .108). A considerable heterogeneity across studies was revealed (*Q* = 168.99; *P* < .01; *I*^2^ = 99.4%; τ^2^ = 3.57). One study (30 participants)^31^ evaluated the heaviness intensity score after 6 months of treatment and showed a significant decrease of −1.9 cm (95% CI, −2.1 to −1.7; *P* < .001) (Supplementary Table IV, online only).

#### Leg cramps

**Fig 4:**
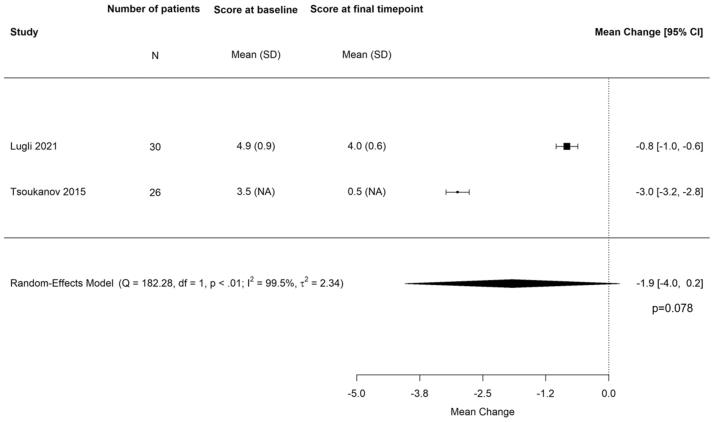
Leg cramp symptom. Forest plot showing the pooled mean change (*MC*) of cramp score intensity from baseline to the last postbaseline value. Error bars represent 95% confidence interval (*CI*). *df*, degrees of freedom; *I*^*2*^, *I*-squared; *N*, number; *NA*, not available; *Q*, Cochrane’s *Q* test statistics; *RE*, random effect; *SD*, standard deviation; *τ*^*2*^, tau-squared.

Leg cramp score was reported in two single-arm studies (56 MPFF-treated participants).^31^^,^^32^ A decrease was observed in cramp intensity score with a MC of −1.9 cm (95% CI, −4.0 to 0.2), without reaching significance (*P* = .078). A considerable heterogeneity across studies was observed (*Q* = 182.28; *P* < .01; *I*^2^ = 99.5%; τ^2^ = 2.34) (Fig 4, Supplementary Tables IV and IX, online only).

Complete resolution of cramp symptoms was reported in two studies (77 participants).^33^^,^^34^ A statistically significant percentage of participants experienced resolution of cramps, 97.8% (95% CI, 83.3-100%; *P* < .001), with a moderate heterogeneity across studies (*Q* = 5.12; *P* = .02; *I*^2^ = 80.5%; τ^2^ = 0.03) (Supplementary Table X, online only).

Subgroup analysis by treatment duration (Supplementary Table IV, online only) showed that cramp intensity decreased by −3.0 cm (95% CI, −3.2 to −2.8) in the group of participants treated for 1 to 2 months (one study^32^), −0.4 cm (95% CI, −0.6 to −0.2) in the group treated for 3 to 5 months (one study^31^), and −0.8 cm (95% CI, −1.0 to −0.6) in the group treated for 6 months or more (one study^31^) (all *P* < .001).

### Quality of life

**Fig 5:**
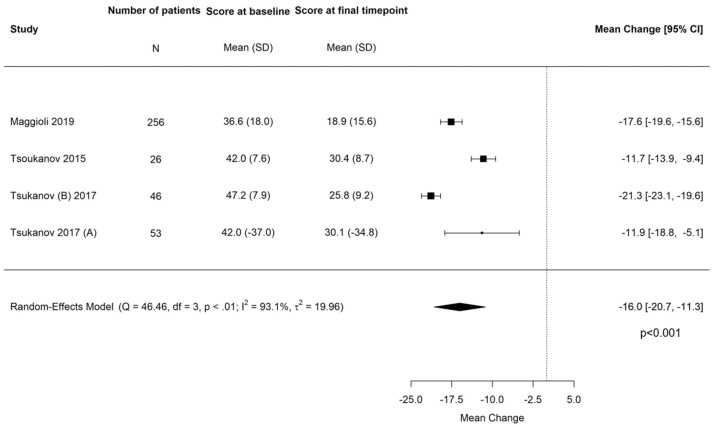
Evolution of quality of life (*QoL*). Forest plot showing the pooled mean change (*MC*) of QoL from baseline to the last postbaseline value. Error bars represent 95% confidence interval (*CI*). *df*, degrees of freedom; *I*^*2*^, *I*-squared; *N*, number; *Q*, Cochrane’s *Q* test statistics; *RE*, random effect; *SD*, standard deviation; *τ*^*2*^, tau-squared.

Four studies^30^^,^^32^, ^33^, ^34^ reported data from CIVIQ-20 expressed as GIS. Overall, data from 381 MPFF-treated patients showed a significant improvement in QoL with a MC of −16.0 points (95% CI, −20.7 to −11.3; *P* < .001), with considerable heterogeneity across studies (*Q* = 46.46; *P* < .001; *I*^2^ = 93.15%; τ^2^ = 19.96) (Fig 5, Supplementary Table XI, online only).

## Discussion

### Summary of findings

This meta-analysis provides comprehensive data on the effectiveness of MPFF in alleviating symptoms, such as leg pain, heaviness, and cramps, and enhancing QoL in patients in the early stages of CVD. The analysis includes data from five prospective studies (one RCT and four non-RCTs), encompassing a total of 411 participants graded C0s-C1 receiving MPFF 1000 mg daily.

A significant improvement in leg symptoms was observed in this analysis. Specifically, pain and heaviness intensity scores were significantly reduced. In addition, complete resolution of these symptoms was observed in a substantial proportion of participants over the period of observation. Tsukanov’s studies conducted in female patients with transient reflux reported the complete disappearance of leg pain symptoms in all (100.0%) participants, whereas heaviness symptoms were completely resolved in 87.9%.^33^^,^^34^ Given the significant positive relationship between superficial reflux and heaviness in females,^35^ it is not surprising that MPFF treatment, which suppresses transitory reflux in at least 92.5% of patients, is associated with the disappearance of heaviness in 88.6% of patients and of venous pain in all patients who reported it.^33^ However, these findings should be interpreted with caution given the open-label design and the population studied, as these factors may introduce bias and limit the robustness of the results. Although the reduction in cramp intensity score observed in this analysis did not reach statistical significance because of heterogeneity among the studies,^31^^,^^32^ complete resolution of cramp symptoms was observed in 97.8% of the participants.^33^^,^^34^ Improvement in symptoms also translated into enhanced QoL, as reflected by a significant reduction in GIS assessed by the CIVIQ questionnaires. The observed alignment between symptom relief and QoL improvements suggests a potential clinical benefit of MPFF treatment in improving both physical comfort and overall well-being in patients with early stage venous disease.

The population included in the present meta-analysis comprised a large proportion of female participants (96.6%). This is consistent with the literature showing that telangiectasia, reticular veins, and varicose veins categorized as C1-C2 are more commonly reported in women, whereas men often present with more advanced stages, such as skin changes and ulcers.^36^ One possible explanation is that women tend to seek medical attention earlier because of aesthetic concerns. In the Vein Consult Program, while the percentage of women under 35 years of age classified as C0 was higher than that of men, this trend reversed after the age of 35, with a greater percentage of men classified as C0.^9^ A significant factor responsible for the sex-specific differences in the prevalence of the disease is the hormonal influence on women during menstrual cycles and pregnancies that leads to changes in venous hemodynamic, flow disturbances, and contributes to the inflammatory reaction responsible for symptoms.^37^

Although the early stages of the disease are often overlooked by health care professionals, patients in the C0s-C1 stages experience a high prevalence of symptoms that may significantly disrupt their daily activities and overall lifestyle. Notably, their QoL may be negatively impacted. In the Vein Alarm Study, a positive correlation was found between QoL and disease severity starting at the very early stage of the disease (C0s).^38^ These CVD symptoms may be exacerbated after prolonged periods of standing or sitting. The prevalence of symptoms in these subjects was assessed in the Acireale Project conducted in Italy between 1989 and 1992. The study reported heavy legs (74.4%), followed by nocturnal cramps and restless leg syndrome (29.3%) in C0s patients.^39^ More recently, VEINSTEP, a study including 6236 participants, confirmed that in a real-world setting, CVD symptoms were prevalent across all CEAP class, including C0s. The mean number of symptoms and their global severity score, as well as the QoL score, increased with the CEAP classes.^40^ Other studies have similarly reported a positive correlation between the percentage of patients with venous symptoms and CEAP class, as well as between the severity score and CEAP classes.^41^^,^^42^ In contrast, in the Edinburgh Vein Study, no correlation was observed between the occurrence of symptoms and the presence of varicose veins or venous reflux.^7^ This absence of correlation was also observed in a study by Darvall et al,^43^ who demonstrated that lower-limb symptoms significantly impacted QoL regardless of the disease's clinical stage.

The existence of C0s patients with venous symptoms despite the absence of detectable anatomical or morphological changes, as defined by functional CVD, is now well accepted, even if the exact pathophysiological pathway remains incompletely elucidated.^44^^,^^45^ Indeed, venous hemodynamics and venous volume changes, which are the primary signs of CVD, are not easy to detect by routine ultrasound investigation commonly used in daily practice.^46^ In the 1970s, Bassi et al^47^ proposed the term *functional phlebopathy* to describe these symptomatic patients presenting with abnormal venous function in the absence of anatomic alteration. Between 1989 and 1992, Andreozzi et al found that nearly half of C0s patients showed no evidence of venous reflux on duplex scanning but exhibited abnormal venous compliance when assessed by plethysmography. The authors termed this reduction in venous wall tone hypotonic phlebopathy.^39^^,^^45^ In 2011, Vincent et al^48^ found that valvular incompetence from the second to sixth generation of tributaries of the GSV could be observed in C0 and C1 lower limbs, despite no observation of GSV truncal reflux. These results were corroborated by Lugli et al^49^ who hypothesized that C0s venous symptoms may originate from the second to sixth generation of saphenous tributaries and small veins that are not connected to the saphenous system. Their results revealed a significantly higher bidirectional flow in C0s patients compared with controls, suggesting the presence of reflux in nonaxial veins.^49^ These findings underscore the presence of venous disturbances at the level of small veins and microcirculation, which may be key contributors to the symptoms experienced by C0s patients even in the absence of detectable venous reflux or other large vessel abnormalities.

Although the sequence of events in CVD pathogenesis is not fully understood, it is accepted that venous inflammation because of hydrostatic pressure-induced shear stress disturbances is the cornerstone of pathology. This leads to molecular and cellular processes responsible for the vascular wall, as well as microvalve damage that once initiated, perpetuates itself, worsening existing venous hypertension.^2^^,^^50^ Recent studies support this hypothesis, emphasizing that remodeling in the venous wall is a continuous physiological process that when dysregulated can lead to early varicosity development. A key factor in this dysregulation is the limited ability of the human body to adapt to prolonged standing and sitting, which is increasingly prevalent in modern industrialized societies.^51^ A possible mechanism to explain the pain and heaviness experienced in the early stage of CVD could be the increase in venous wall tension because of increased hydrostatic pressure. In the early stages of the disease, before venous remodeling occurs, venous distensibility is limited. This reduced distensibility in these early stages leads to increased venous wall tension, which can cause pain. The elevated pressure can also compress the vasa vasorum which supply oxygen to the venous wall, causing localized hypoxia. Hypoxia in the venous wall triggers a cascade of inflammatory responses, releasing cytokines and other mediators that sensitize nerve endings, resulting in pain. This inflammatory response, coupled with venous wall tension, contributes to heaviness and discomfort often reported by patients.^52^ Restless legs and cramps may be linked to hemorheological issues, such as increased red blood cell hyperviscosity and hyperaggregation, which impair microcirculation.^53^ Once again, these blood flow abnormalities can restrict oxygen delivery leading to localized hypoxia. These mechanisms may explain the muscle cramps and sensations of restlessness in the legs, particularly during periods of low physical activity, such as nighttime or prolonged sitting.^52^

Early detection of CVD using ultrasound but also other more specific techniques such as plethysmography is crucial to diagnose patients and initiate optimal treatment not only to alleviate symptoms but also to potentially prevent or delay the progression of the disease. Indeed, these patients may evolve toward more severe stages including edema, skin changes, and venous ulceration. Clinical worsening and progression to a more advanced stage were observed in 57.8% of patients with varicose vein alone over a period of 13.4 years. One-third (31.9%) of adults with isolated varicose veins at baseline developed signs of CVI such as edema or skin changes, and in almost all patients, the condition deteriorated when both varicose veins and CVI were present.^7^ The rate of disease progression is influenced by several key factors, including genetics, lifestyle habits such as physical inactivity and elevated BMI, which contribute to increased venous pressure and vascular dysfunction.^2^ Recent evidence suggests that CVD is independently associated with the presence of cardiovascular disease, and this association increases proportionally along with higher CEAP stages.^12^ In addition to the coexistence of both diseases, CVD might be independently associated with an increased risk of major adverse cardiovascular events^13^ as well all-cause mortality.^12^ This underscores a potential crosstalk between veins and arteries, positioning venous disease as part of the cardiovascular continuum and raises the need for increased physician awareness to assess CVD at its earliest stages.

Management of these patients as early as possible is thus important. Therapeutic options involve a comprehensive approach that includes lifestyle modifications, pharmacological treatment with VADs, and the use of compression therapy alone or in addition to venous procedural interventions such as sclerotherapy or endovenous treatment. Unfortunately, no longitudinal study has been performed to evaluate the effects of these treatments on disease progression. Experimental evidence indicated that MPFF improved venous tone^54^ and reduced inflammation within the venous walls as well as at the microvalve level suggesting a possible protective role.^55^^,^^56^ Clinical studies have shown that MPFF treatment decreased the number of microvalve sites with reflux,^31^ reversed evening (situational) reflux of C0s women,^32^ and reduced the evening diameter of GSV.^32^ These benefits support MPFF endorsement in the clinical guidelines for the management of symptoms and signs in all patients with CVD, including those in the early stages.^14^^,^^15^

MPFF is also used as an adjunct to venous procedural intervention in patients with reticular veins or varicose veins, including sclerotherapy, surgery, or endovenous treatment.^15^ Patients who had undergone such procedures were excluded from the current study to ensure the integrity of the results. Interestingly, the VEIN ACT PROLONGED-C1 study, which evaluated the addition of MPFF in C1 patients with dilated intradermal veins scheduled for sclerotherapy, found that resolution of venous symptoms after sclerotherapy was significantly greater in C1-stage patients treated with MPFF compared with those who underwent sclerotherapy alone.^57^

This meta-analysis found that MPFF was effective in reducing symptom intensity over treatment periods ranging from 2 to 6 months. This indicates that the treatment's effectiveness was potentially sustained over time. However, additional studies are required to evaluate the long-term sustainability of clinical benefit, to explore the kinetics of symptom reappearance or disappearance after treatment discontinuation, potential plateau effects, as well as the need for treatment adjustment over time. MPFF was also significantly effective across different study designs, including both non-RCTs and RCTs. A greater symptom intensity reduction was noted in the RCTs compared with the non-RCTs.

### Limitations

The main limitation of this analysis was its low number of studies and sample size. This limited the power of some analyses and the ability to perform metaregression or subgroup analysis as well as assessment of the publication bias. In addition, smaller studies tend to report greater intervention effects than larger studies, which may introduce bias.^58^ The substantial heterogeneity observed across studies precluded conducting a powerful meta-analysis. Notably, all included studies (RCTs and non-RCTs) did not have a control arm. Among the included studies, three non-RCTs^32^, ^33^, ^34^ enrolled only females resulting in a lack of some information from male participants and may limit the generalizability of the results. Although the study was funded by Servier, bias was minimized using predefined protocols, PROSPERO registration, and the independent selection of studies, as well as the extraction and analysis of data by an external contract research organization. Despite these safeguards, some influence cannot be fully excluded. Considering the limitations of our analysis, the results should be interpreted with caution.

### Strengths

None of the studies included in this analysis allowed compression therapy or concomitant treatment that may have jeopardized the results. Therefore, the results observed in this work may be allocated solely to the treatment with MPFF. By targeting the early stages of the disease, this research addresses a gap in the existing literature and provides valuable insights that were previously unexplored or only in scarce studies with low sample size. Despite the limited number of studies available, this work offers an overall view of the MPFF treatment effect in relieving CVD symptoms and improving QoL based on RCTs and non-RCTs in C0s and C1 CVD patients. We acknowledge that symptoms and QoL are inherently subjective parameters. However, some of the studies included in this analysis provide complementary objective data. For instance, Tsukanov et al^32^, ^33^, ^34^ reported that treatment with MPFF was associated with normalization of GSV diameter and resolution of situational reflux, whereas Lugli et al^31^ observed significant reductions over time in the number of microvalve sites exhibiting reflux. Although these findings may suggest a potential reversibility of certain pathophysiological features of CVD in early stages, further studies incorporating objective quantitative measures, such as hemodynamic assessments or relevant biological biomarkers, are needed to provide a more comprehensive evaluation of treatment effects. In addition, this analysis highlights the effectiveness of MPFF both in the initial phase of treatment and over time in this specific population, strengthening the evidence for long-term treatment. However, since only one study has assessed effectiveness up to 6 months,^31^ additional research is needed to evaluate long-term outcomes.

## Conclusions

This meta-analysis adds to the evidence supporting the effectiveness of MPFF treatment in relieving venous leg symptoms and improving QoL in patients with early stage CVD. Improvements in symptoms were observed throughout the 6-month observation period, suggesting a sustained effect. Although further long-term studies on C0s-C1 patients are needed to assess the potential role of early intervention in preventing disease progression and mitigating long-term vascular damage, these data highlight the clinical value of initiating MPFF treatment at the earliest stage of the disease to provide symptom relief and improve health-related QoL.

## Author contributions

The authors meet the International Committee of Medical Journal Editors criteria for authorship for this article and take responsibility for the integrity of the work. Study conception and design were performed by all authors; literature review, data collection, and analysis were performed by V.B.G., M.B.O., and S.S. All authors participated in data interpretation and article preparation. All authors read and approved the final version of the article.

## Availability of data

The study data set is available from the corresponding author on reasonable request.

## Funding

This work was sponsored by 10.13039/501100011725Servier. Article processing charges (and the open access fee) were funded by 10.13039/501100011725Servier. Soladis Clinical studies and Soladis received fees from Servier for conducting this work and for providing medical writing assistance.

## Disclosures

A. Mansilha received research support from Servier, Alfa Sigma, and OM Pharma. I. Zolotukhin has served as a speaker for Servier, Alfa Sigma, and Innotech. V. Blanc-Guillemaud and H.P. Yaltirik are employees of Servier Medical Affairs, France. S. Serifou is an employee of Soladis. M.-B. Onselaer is an employee of Soladis Clinical Studies. E. Bouskela, M. Amore, A. Ministro, and M. Sirlak declare no conflicts of interest.

## Figures and Tables

**Supplementary Fig (online only):**
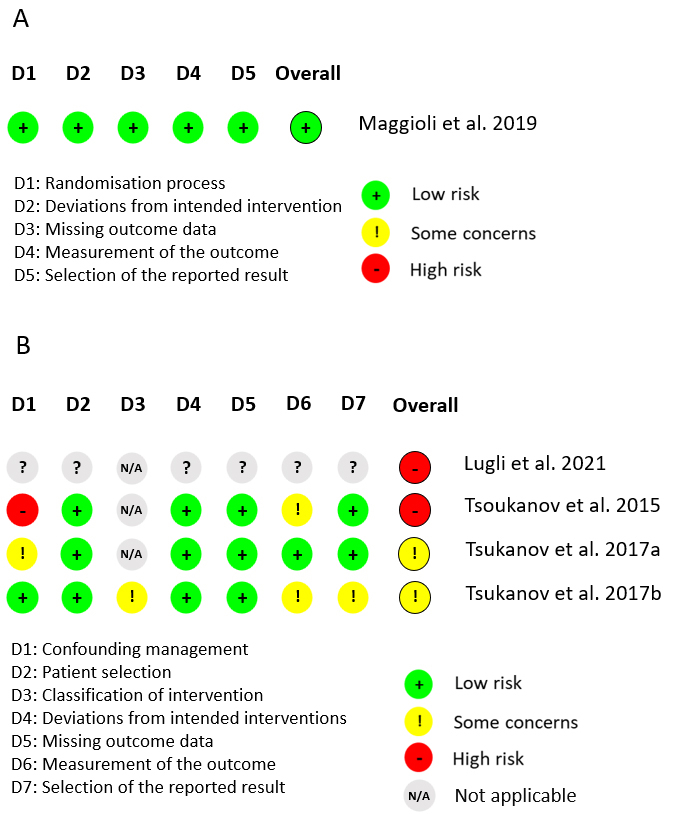
Risk of bias (RoB) assessment. **A,** Randomized controlled trial according to RoB2 tool and **(B)** nonrandomized controlled trials (ie, comparative, single-arm, and observational studies) according to the ROBINS-I tool included in the analysis.

## Appendix (online only)

Supplementary Tables I-XI (online only)
