# Supplementary material for: Effectiveness of micronized purified flavonoid fraction in early stage chronic venous disease—Clinical, Etiological, Anatomical, and Pathophysiological C0s-C1: A systematic review and meta-analysis
Source: J Vasc Surg Venous Lymphat Disord. 2026 Jun 22;14(5):102558. doi: 10.1016/j.jvsv.2026.102558 (PMC13393450; doi:10.1016/j.jvsv.2026.102558)
Supplement: Supplementary Tables I-XI (online only) [file mmc1.docx]

**Effectiveness of micronized purified flavonoid fraction in early-stages chronic venous disease – CEAP C0s/C1**: **A systematic review and meta-analysis.**

TABLES LIST:

**Supplemental Table I**. Search strategy

**Supplemental Table II**. Description of the included studies

**Supplemental Table III**. Patients baseline characteristics

**Supplemental Table IV**. Symptom intensity evolution

**Supplemental Table V.** Characteristics of the studies evaluating the pooled mean change of pain score intensity from baseline to the last post baseline value.

**Supplemental Table VI.** Characteristics of the studies evaluating the proportion of patients with complete resolution of leg pain symptoms.

**Supplemental Table VII.** Characteristics of the studies evaluating the pooled mean change of heaviness score intensity from baseline to the last post baseline value.

**Supplemental Table VIII.** Characteristics of the studies evaluating the proportion of patients with complete resolution of leg heaviness symptoms.

**Supplemental Table IX.** Characteristics of the studies evaluating the pooled mean change of cramp score intensity from baseline to the last post baseline value.

**Supplemental Table X.** Characteristics of the studies evaluating the proportion of patients with complete resolution of leg cramp symptoms.

**Supplemental Table XI.** Characteristics of the studies evaluating the pooled mean change of quality of life from baseline to the last post baseline value.

| The search terms included:  (“CEAP early stage” OR “venous disease early stage” OR “telangiectasia” OR “reticular vein” OR “C0r” OR “C0s” OR “C1” OR “C0s-C1”)  AND  (“diosmin” AND “hesperidin”) OR “MPFF” OR “micronised purified flavonoid fraction” OR “micronized purified flavonoid fraction” OR “Alvenor” OR “Ardium” OR “Arvenum” OR “Capiven” OR “Daflon” OR Detralex OR “Elatec” OR “Gevoxum” OR “Variton” OR “Venitol” OR “Venixxa”)  AND  (“venous disease” OR “vein disease” OR “venous incompetence” OR “vein incompetence” OR “venous disorder” OR “vein disorder” OR “venous reflux” OR “vein reflux” OR “venous symptoms” OR “vein symptoms”) |
| --- |

| **Study name** | **Country** | **Study design** | **Compa-rator** | **Duration** | **CEAP** | **CEAP (N)** | **C0s (%)** | **C1**  **(%)** | **Etiology** | **Included  (N)** | **Arm  Description** | **MPFF treated  (N)** |
| --- | --- | --- | --- | --- | --- | --- | --- | --- | --- | --- | --- | --- |
| Lugli 2021 | Italy | Single arm | None | 6 months | C0, C1 | 30 | 9.0 | 81.0 | primary | 30 | MPFF 1000mg DIE | 30 |
| Maggioli 2019 | Argentina, Brazil, Czech Republic, Malaysia, Romania, Russia, Slovakia, Slovenia, Spain, Thailand, Turkey and Vietnam | Post-Hoc RCT | Other Regimen | 8 weeks | C0, C1 | 256 | 3.5 | 96.5 | primary | 256 | MPFF 1000mg DIE | 122 |
|  |  |  |  |  |  |  |  |  |  |  | MPFF 500mg BID | 134 |
|  |  |  |  |  |  |  |  |  |  |  | Pooled | 256 |
| Tsoukanov 2015 | Russia | Single arm | None | 2 months | C0 | 41 | 100.0 | 0.0 | ND | 41 | MPFF 1000mg DIE | 26* |
| Tsukanov 2017 (A) | Russia | Single arm | None | 3 months | C1 | 96 | 0.0 | 100.0 | primary | 96 | MPFF 1000mg DIE | 53* |
| Tsukanov 2017 (B) | Russia | Cohorts | None | 3 months | C0-C2 | 106 | 51.9 | 48.1 | primary | 294 | C1 vs C2  MPFF 1000mg DIE | 56* |
| **TOTAL C0s/C1, N (%)** | |  |  |  |  | **529** | **108 (20)** | **421 (80)** |  |  |  |  |

*Only C0s-C1 patients with evening reflux and treated with MPFF were included in the analysis. BID, twice a day; CEAP, clinical, etiological, anatomical and pathophysiological; DIE, once daily; MPFF, micronized purified flavonoid fraction; N, number of patients; ND, not determined; RCT, randomized controlled trial.

| **Study ID** | **Design** | **Arm Description** | **Age**  **(N)** | **Age, years  Mean (SD)** | **Sex**  **(N)** | **Female  (%)** | **BMI (N)** | **BMI, kg/m^2^  Mean (SD)** |
| --- | --- | --- | --- | --- | --- | --- | --- | --- |
| Lugli 2021 | Non-RCT-Single arm | MPFF 1000mg DIE | 30 | 51.3 (9) | 30 | 96.7 | 30 | 24 (3) |
| Maggioli 2019 | RCT-Post Hoc analysis | MPFF 500mg BID  /MPFF 1000mg DIE | 256 | 41.4 (11.3) | 256 | 93.4 | 256 | 24.1 (3.1) |
| Tsoukanov 2015 | Non-RCT-Single arm | MPFF 1000mg DIE | 41 | 35.4 (15.1) | 41 | 100.0 | ND | ND |
| Tsukanov 2017 (A) | Non-RCT-Single arm | MPFF 1000mg DIE | 96 | 31 (4.4) | 96 | 100.0 | ND | ND |
| Tsukanov 2017 (B) | Non-RCT-Open label study | MPFF 1000mg DIE | 294 | 41.5 (NA) | 294 | 100.0 | ND | ND |
| **TOTAL** |  |  | **717** | **40.1 (8.0)** | **529** | **96.6** | **286** | **23.9 (3.1)** |

BID, twice a day; BMI, body mass index; DIE, once a day; MPFF, micronized purified flavonoid fraction; N, number of patients; NA, not available; ND, not determined; RCT, randomized controlled trial; SD, standard deviation.

| **CVD symptoms** | **Analysis** | **Number of studies**  **(N)** | **Number of patients**  **(N)** | **MC in cm**  **(95% CI)** | **P-Value** | **I²** | **τ²** |
| --- | --- | --- | --- | --- | --- | --- | --- |
| Pain | Overall | 3 | 312 | -2.3 (-3.1; -1.6) | <0.001 (S) | 94.0% | 0.45 |
|  | Sensitivity analysis* | - | - | - | - | - | - |
|  | RCTs | 1 | 256 | -3.1 (-3.4; -2.8) | <0.001 (S) | - | - |
|  | Non-RCTs | 2 | 56 | -1.9 (-2.2; -1.7) | <0.001 (S) | 0.0% | 0.00 |
|  | Short term (≤2 months) | 2 | 282 | -2.6 (-3.7; -1.4) | <0.001 (S) | 93.6% | 0.65 |
|  | Medium term (3 to 5 months) | 1 | 30 | -1.3 (-1.6; -1.1) | <0.001 (S) | - | - |
|  | Long term (≥6 months) | 1 | 30 | -1.9 (-2.2; -1.7) | <0.001 (S) | - | - |
| Heaviness | Overall | 4 | 358 | -2.8 (-3.5; -2.1) | <0.001 (S) | 95.2% | 0.49 |
|  | Sensitivity analysis* | 2 | 302 | -3.3 (-3.6; -3.0) | <0.001 (S) | 38.9% | 0.02 |
|  | RCTs | 1 | 256 | -3.2 (-3.5; -2.9) | <0.001 (S) | - | - |
|  | Non-RCTs | 3 | 102 | -2.6 (-3.6; -1.7) | <0.001 (S) | 95.7% | 0.62 |
|  | Short term (≤2 months) | 2 | 282 | -2.9 (-3.6; -2.2) | <0.001 (S) | 84.3% | 0.21 |
|  | Medium term (3 to 5 months) | 2 | 76 | -2.2 (-4.8; 0.5) | 0.108 (NS) | 99.4% | 3.57 |
|  | Long term (≥6 months) | 1 | 30 | -1.9 (-2.1; -1.7) | <0.001 (S) | - | - |
| Cramps | Overall | 2 | 56 | -1.9 (-4.0; 0.2) | 0.078 (NS) | 99.5% | 2.34 |
|  | Sensitivity analysis* | 0 | - | - | - | - | - |
|  | RCTs | 0 | - | - | - | - | - |
|  | Non-RCTs | 2 | 56 | -1.9 (-4.0; 0.2) | 0.078 (NS) | 99.5% | 2.34 |
|  | Short term (≤2 months) | 1 | 26 | -3.0 (-3.2; -2.8) | <0.001 (S) | - | - |
|  | Medium term (3 to 5 months) | 1 | 30 | -0.4 (-0.6; -0.2) | <0.001 (S) | - | - |
|  | Long term (≥6 months) | 1 | 30 | -0.8 (-1.0; -0.6) | <0.001 (S) | - | - |

* Sensitivity analysis excluded the studies assessed with high risk of bias. CI, confidence interval; I², I squared; MC, mean change; N, number; NS, not significant; RCT, randomized controlled trial; S, significant; τ², tau squared.

| **Study ID** | **Scale range** | **Weights (%)** | **N** | **Baseline Mean (SD)** | **Post-baseline value (Week)** | **Post-baseline value Mean (SD)** | **Mean Change (SD)** | **Mean Change [95% CI]** | **P-value (Significance)** |
| --- | --- | --- | --- | --- | --- | --- | --- | --- | --- |
| Lugli 2021 | 0-10 | 34.6% | 30 | 5.5 (1.0) | 24 | 3.5 (0.5) | -1.9 (0.7*) | -1.9 [-2.2; -1.7] |  |
| Maggioli 2019 | 0-10 | 34.0% | 256 | 6.2 (1.8) | 8 | 3.0 (2.1) | -3.1 (2.5) | -3.1 [-3.4; -2.8] |  |
| Tsoukanov 2015 | 0-10 | 31.4% | 26 | 2.7 (NA) | 8 | 0.8 (NA) | -2.0 (1.3**) | -2.0 [-2.4; -1.5] |  |
| **Random effect model** |  | **100%** |  |  |  |  |  | **-2.3 [-3.1; -1.6]** | **< 0.001 (S)** |
| Measure of the model used: Mean Change   Heterogeneity, Q = 37.99, df = 2 (P = < 0.001 (S)); I^2^ = 93.95%, τ^2^ = 0.45 | | | | | | | | | |
| Fixed effect model   Estimate = -2.4 [-2.5; -2.2]   P-value (Significance): < 0.001 (S) | | | | | | | | | |
| *: Derived from baseline and postbaseline timepoints, with assumed correlation Rho = 0.75 between timepoints | | | | | | | | | |
| **: Imputed SD from mean SD across all studies using the same scale | | | | | | | | | |

CI: confidence interval, df: degrees of freedom, ID: identification, I^2^: I-squared, N: number of patients with available data, NA: not available,
Q: Cochrane’s Q test statistics, S: significant, SD: standard deviation, τ^2^: tau-squared.

| **Study ID** | **Weights (%)** | **Post-baseline value (Week)** | **Baseline Number of patients with symptoms  (N)** | **Post-baseline Number of patients with resolved symptoms  (N)** | **Proportion of resolution (%) [95% CI]** | **P-value (Significance)** |
| --- | --- | --- | --- | --- | --- | --- |
| Tsukanov (A) 2017 | 34.1% | 12 | 15 | 15 | 100.0 [93.7; 100.0] |  |
| Tsukanov (B) 2017 | 31.8% | 12 | 14 | 14 | 100.0 [93.3; 100.0] |  |
| **Random effect model** | **100%** |  |  |  | **100.0 [96.7; 100.0]** | **< 0.001 (S)** |
| Measure of the model used: Arcsine square root transformed proportion   Heterogeneity, Q = 0.00, df = 2 (P = 1.000 (NS)); I^2^ = 0.00%, τ^2^ = 0.00 | | | | | | |
| Fixed effect model   Estimate = 100.0 [96.7; 100.0]   P-value (Significance): < 0.001 (S) | | | | | | |

CI: confidence interval, df: degrees of freedom, ID: identification, I^2^: I-squared, N: number of patients with available data, Q: Cochrane’s Q test statistics, S: significant, τ^2^: tau-squared.

| **Study ID** | **Scale range** | **Weights (%)** | **N** | **Baseline Mean (SD)** | **Post-baseline value (Week)** | **Post-baseline value Mean (SD)** | **Mean Change (SD)** | **Mean Change [95% CI]** | **P-value (Significance)** |
| --- | --- | --- | --- | --- | --- | --- | --- | --- | --- |
| Lugli 2021 | 0-10 | 25.9% | 30 | 5.4 (0.9) | 24 | 3.5 (0.5) | -1.9 (0.6*) | -1.9 [-2.1; -1.7] |  |
| Maggioli 2019 | 0-10 | 25.3% | 256 | 6.3 (1.8) | 8 | 3.1 (2.0) | -3.2 (2.4) | -3.2 [-3.5; -2.9] |  |
| Tsoukanov 2015 | 0-10 | 23.8% | 26 | 4.5 (NA) | 8 | 2.0 (NA) | -2.5 (1.2**) | -2.5 [-3.0; -2.0] |  |
| Tsukanov (B) 2017 | 0-10 | 24.9% | 46 | 5.2 (NA) | 12 | 1.7 (NA) | -3.5 (1.2**) | -3.5 [-3.8; -3.2] |  |
| **Random effect model** |  | **100%** |  |  |  |  |  | **-2.8 [-3.5; -2.1]** | **< 0.001 (S)** |
| Measure of the model used: Mean Change   Heterogeneity, Q = 80.87, df = 3 (P = < 0.001 (S)); I^2^ = 95.17%, τ^2^ = 0.49 | | | | | | | | | |
| Fixed effect model   Estimate = -2.6 [-2.7; -2.4]   P-value (Significance): < 0.001 (S) | | | | | | | | | |
| *: Derived from baseline and postbaseline timepoints, with assumed correlation Rho = 0.75 between timepoints | | | | | | | | | |
| **: Imputed SD from mean SD across all studies using the same scale | | | | | | | | | |

CI: confidence interval, df: degrees of freedom, ID: identification, I^2^: I-squared, N: number of patients with available data, NA: not available,
Q: Cochrane’s Q test statistics, S: significant, SD: standard deviation, τ^2^: tau-squared.

| **Study ID** | **Weights (%)** | **Post-baseline value (Week)** | **Baseline Number of patients with symptoms  (N)** | **Post-baseline Number of patients with resolved symptoms  (N)** | **Proportion of resolution (%) [95% CI]** | **P-value (Significance)** |
| --- | --- | --- | --- | --- | --- | --- |
| Tsukanov (A) 2017 | 33.5% | 12 | 53 | 47 | 86.8 [76.5; 94.5] |  |
| Tsukanov (B) 2017 | 29.1% | 12 | 46 | 41 | 89.1 [78.6; 96.4] |  |
| **Random effect model** | **100%** |  |  |  | **87.9 [80.8; 93.6]** | **< 0.001 (S)** |
| Measure of the model used: Arcsine square root transformed proportion   Heterogeneity, Q = 0.13, df = 1 (P = 0.721 (NS)); I^2^ = 0.00%, τ^2^ = 0.00 | | | | | | |
| Fixed effect model   Estimate = 87.9 [80.8; 93.6]   P-value (Significance): < 0.001 (S) | | | | | | |

CI: confidence interval, df: degrees of freedom, ID: identification, I^2^: I-squared, N: number of patients with available data, Q: Cochrane’s Q test statistics, S: significant, τ^2^: tau-squared.

| **Study ID** | **Scale range** | **Weights (%)** | **N** | **Baseline Mean (SD)** | **Post-baseline value (Week)** | **Post-baseline value Mean (SD)** | **Mean Change (SD)** | **Mean Change [95% CI]** | **P-value (Significance)** |
| --- | --- | --- | --- | --- | --- | --- | --- | --- | --- |
| Lugli 2021 | 0-10 | 50.0% | 30 | 4.9 (0.9) | 24 | 4.0 (0.6) | -0.8 (0.6*) | -0.8 [-1.0; -0.6] |  |
| Tsoukanov 2015 | 0-10 | 50.0% | 26 | 3.5 (NA) | 8 | 0.5 (NA) | -3.0 (0.6**) | -3.0 [-3.2; -2.8] |  |
| **Random effect model** |  | **100%** |  |  |  |  |  | **-1.9 [-4.0; 0.2]** | **0.078 (NS)** |
| Measure of the model used: Mean Change   Heterogeneity, Q = 182.28, df = 1 (P = < 0.001 (S)); I^2^ = 99.45%, τ^2^ = 2.34 | | | | | | | | | |
| Fixed effect model   Estimate = -1.8 [-2.0; -1.7]   P-value (Significance): < 0.001 (S) | | | | | | | | | |
| *: Derived from baseline and postbaseline timepoints, with assumed correlation Rho = 0.75 between timepoints | | | | | | | | | |
| **: Imputed SD from mean SD across all studies using the same scale | | | | | | | | | |

CI: confidence interval, df: degrees of freedom, ID: identification, I^2^: I-squared, N: number of patients with available data, NA: not available,
Q: Cochrane’s Q test statistics, S: significant, SD: standard deviation, τ^2^: tau-squared.

| **Study ID** | **Weights (%)** | **Post-baseline value (Week)** | **Baseline Number of patients with symptoms  (N)** | **Post-baseline Number of patients with resolved symptoms  (N)** | **Proportion of resolution (%) [95% CI]** | **P-value (Significance)** |
| --- | --- | --- | --- | --- | --- | --- |
| Tsukanov (A) 2017 | 38.5% | 12 | 53 | 50 | 92.5 [83.9; 97.9] |  |
| Tsukanov (B) 2017 | 31.5% | 12 | 24 | 24 | 100.0 [96.1; 100.0] |  |
| **Random effect model** | **100%** |  |  |  | **97.8 [83.3; 100.0]** | **< 0.001 (S)** |
| Measure of the model used: Arcsine square root transformed proportion   Heterogeneity, Q = 5.12, df = 1 (P = 0.024 (S)); I^2^ = 80.46%, τ^2^ = 0.03 | | | | | | |
| Fixed effect model   Estimate = 96.4 [91.1; 99.4]  P-value (Significance): < 0.001 (S) | | | | | | |

CI: confidence interval, df: degrees of freedom, ID: identification, I^2^: I-squared, N: number of patients with available data, Q: Cochrane’s Q test statistics, S: significant, τ^2^: tau-squared.

| **Study ID** | **Scale range** | **Weights (%)** | **N** | **Baseline Mean (SD)** | **Post-baseline value (Week)** | **Post-baseline value Mean (SD)** | **Mean Change (SD)** | **Mean Change [95% CI]** | **P-value (Significance)** |
| --- | --- | --- | --- | --- | --- | --- | --- | --- | --- |
| Maggioli 2019 | CIVIQ-20 (GIS) | 22.4% | 256 | 36.6 (18.0) | 8 | 18.9 (15.6) | -17.6 (16.2) | -17.6  [-19.6; -15.6] |  |
| Tsoukanov 2015 | CIVIQ-20 (GIS) | 22.1% | 26 | 42.0 (7.6) | 8 | 30.4 (8.7) | -11.7 (5.8*) | -11.7 [-13.9; -9.4] |  |
| Tsukanov (B) 2017 | CIVIQ-20 (GIS) | 22.6% | 46 | 47.2 (7.9) | 12 | 25.8 (9.2) | -21.3 (6.1*) | -21.3 [-23.1; -19.6] |  |
| Tsukanov 2017 (A) | CIVIQ-20 (GIS) | 14.6% | 53 | 42.0 (-37.0) | 12 | 30.1 (-34.8) | -11.9 (25.5*) | -11.9 [-18.8; -5.1] |  |
| **Random effect model** |  | **100%** |  |  |  |  |  | **-16.0 [-20.7; -11.3]** | **< 0.001 (S)** |
| Measure of the model used: Mean Change   Heterogeneity, Q = 46.46, df = 3 (P = < 0.001 (S)); I^2^ = 93.15%, τ^2^ = 19.96 | | | | | | | | | |
| Fixed effect model   Estimate = -17.5 [-18.6; -16.3]  P-value (Significance): < 0.001 (S) | | | | | | | | | |
| *: Derived from baseline and postbaseline timepoints, with assumed correlation Rho = 0.75 between timepoints | | | | | | | | | |
| **: Imputed SD from mean SD across all studies using the same scale | | | | | | | | | |

CI: confidence interval, df: degrees of freedom, ID: identification, I^2^: I-squared, N: number of patients with available data, NA: not available,
Q: Cochrane’s Q test statistics, S: significant, SD: standard deviation, τ^2^: tau-squared.
